# Supplementary material for: Modeling the Wintering Habitat Distribution of the Black Stork in Shaanxi, China: A Hierarchical Integration of Climate and Land Use/Land Cover Data
Source: Animals (Basel). 2023 Aug 27;13(17):2726. doi: 10.3390/ani13172726 (PMC10487094; doi:10.3390/ani13172726)
Supplement: Supplementary file 1 [file animals-13-02726-s001.zip › animals-2555785-supplementary.pdf]

Table S1. Usage and sources of environmental factors used in models

Data type	Environmental variables (Unit)	Modelling usage	Source
Climatic data	Isothermality (-)	SDMs	https://www.worldclim.org/
	Mean temperature of driest quarter (℃)	SDMs	
	Precipitation seasonality (-)	SDMs	
	Precipitation of driest month (mm)	SDMs	
	Annual mean temperature	PLUS	
	Annual precipitation	PLUS	
Natural Environment data	Elevation (m)	SDMs, PLUS	Geospatial Data Cloud http://www. gscloud.cn
	Aspect (°)	SDMs	
	Slope (°)	SDMs	
	Soil type (-)	PLUS	Resource and Environmental Science and Data Center https://www.resdc.cn/
	Distance to river (m)	SDMs	
	LULC (2000, 2010, 2020) (-)	SDMs, PLUS	GLOBELAND30 http://www.globeland30.org/ , https://www.scidb.cn/en
Human interference and socioeconomic data	Population density (persons/km^2)	PLUS	Resource and Environmental Science and Data Center (2015) https://www.resdc.cn/
	GDP (ten thousand Yuan/km^2)	PLUS	
	Nightlight (-)	PLUS	
	Distance to primary road (m)	PLUS	National Geomatic Center of China (2015) http://www.ngcc.cn/ngcc/
	Distance to secondary road (m)	PLUS	
	Distance to highway (m)	SDMs, PLUS	
	Distance to railway (m)	PLUS	
	Distance to prefectural center (m)	PLUS	
	Distance to county center (m)	PLUS	
	Distance to residential area (m)	PLUS	

SDMs: Species Distribution models, PLUS: Patch-generating Land Use Simulation

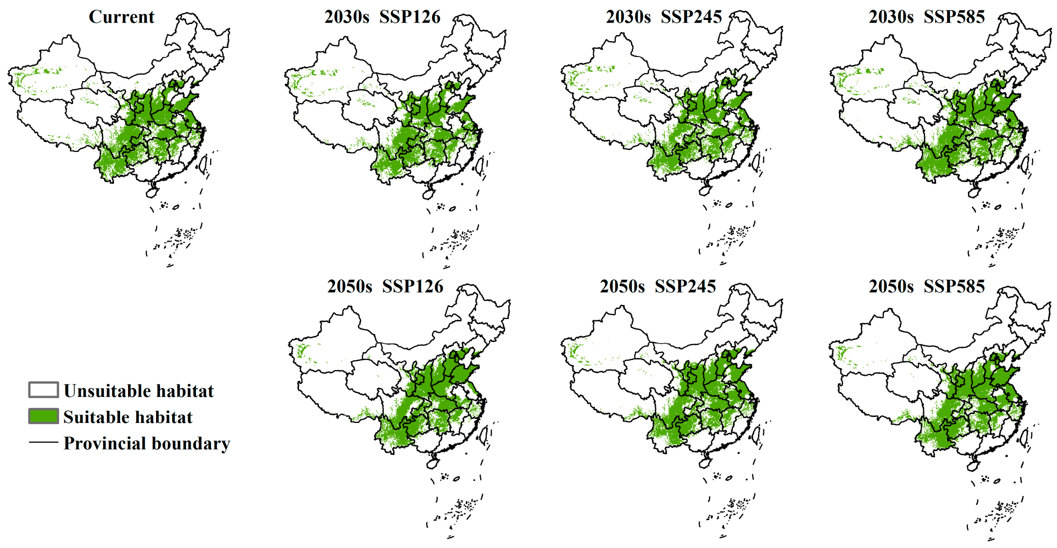

Figure S1. Distribution patterns of suitable habitat for wintering black stork derived from the national model

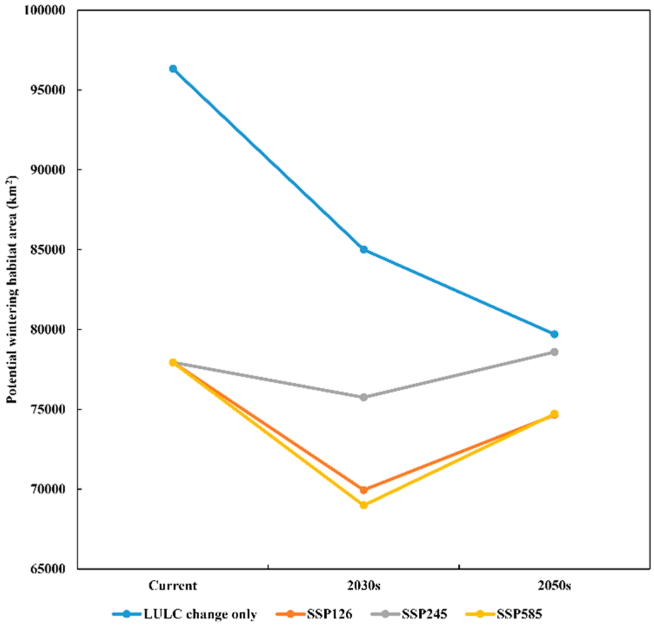

Figure S2. Changes in the area of potential wintering habitat under different future scenarios
